# Leptomeningeal metastasis in breast cancer – a systematic review

**DOI:** 10.18632/oncotarget.5911

**Published:** 2015-11-02

**Authors:** Brian J. Scott, Nancy A. Oberheim-Bush, Santosh Kesari

**Affiliations:** ^1^ Department of Neurology, Lahey Hospital and Medical Center, Burlington, Massachusetts, USA; ^2^ Department of Neurology, University of California, San Francisco, California, USA; ^3^ Translational Neuro-Oncology Laboratories and Department of Neurosciences, Moores UCSD Cancer Center, La Jolla, California, USA

**Keywords:** leptomeningeala metastasis, carcinomatous meningitis, breast cancer, central nervous system metastasis, intrathecal chemotherapy

## Abstract

**Background:**

There is limited data on the impact of specific patient characteristics, tumor subtypes or treatment interventions on survival in breast cancer LM.

**Methods:**

A systematic review was conducted to assess the impact of hormone receptor and HER-2 status on survival in breast cancer LM. A search for clinical studies published between 1/1/2007 and 7/1/2012 and all randomized-controlled trials was performed. Survival data from all studies are reported by study design (prospective trials, retrospective cohort studies, case studies).

**Results:**

A total of 36 studies with 851 LM breast cancer subjects were identified. The majority (87%) were treated with intrathecal chemotherapy. Pooled median overall survival ranged from 14.9-18.1 weeks depending on study type. Breast cancer LM survival (15 weeks) was longer than other solid tumor LM 8.3 weeks and lung cancer LM 8.7 weeks, but shorter than LM lymphoma (15.4 versus 24.2 weeks). The impact of hormone receptor and HER-2 status on survival could not be determined.

**Conclusions:**

A median overall survival of 15 weeks in prospective studies of breast cancer LM provides a historical comparison for future LM breast cancer trials. Other outcomes including the impact of molecular status on survival could not be determined based on available studies.

## INTRODUCTION

The treatment of metastatic breast cancer is increasingly being tailored to specific molecular characteristics and patterns of metastatic spread. Central nervous system (CNS) metastasis occurs in about 5% of those with early stage breast cancer at some point in the course of illness.[1-3] Most often, CNS metastasis occur as a late manifestation of breast cancer, and is accompanied by metastatic spread in other organs. CNS metastasis may less commonly be an early or presenting feature of breast cancer. Understanding and improving upon currently available therapies for CNS metastasis is important, because CNS spread of disease is often a poor prognostic sign. It also frequently has a negative impact on functional status and overall quality of life.

Parenchymal brain metastases (BM) account for the majority (~ 80%) of CNS metastases. Prospective trials have helped to guide treatment decisions for brain metastases.[4] Retrospective reviews have identified factors such as number of metastases, the presence or absence of active systemic disease, and hormone receptor status as having an impact on survival.[5, 6]

Leptomeningeal metastasis (LM) represents a minority of CNS metastasis (11-20%) [2, 7], and there is less data available to inform decisions about therapy. Much of the data is obtained retrospectively. Most studies do not examine breast cancer exclusively, but rather include other solid tumors, hematologic malignancies and primary brain tumors. The direct application of these results to breast cancer is likely to be limited. Since treatment, prognosis, and systemic involvement of each of these cancer types is different, it is logical to consider that LM from breast cancer may have a different natural history and respond differently to treatment than LM from other cancers. In particular, there has been an association between lobular histology and CNS metastasis, and there is some evidence for an increased incidence of brain metastasis in HER-2 + breast cancer.[3] The impact of HER-2 and hormone receptor status in LM is less well-defined. Given the diversity in patient demographics, disease biology and potential therapeutic targets between breast and other cancers, we hypothesized that there would be differences in survival between breast cancer LM and other malignancies, and among breast cancer molecular subtypes.

## RESULTS

**Figure 1:**
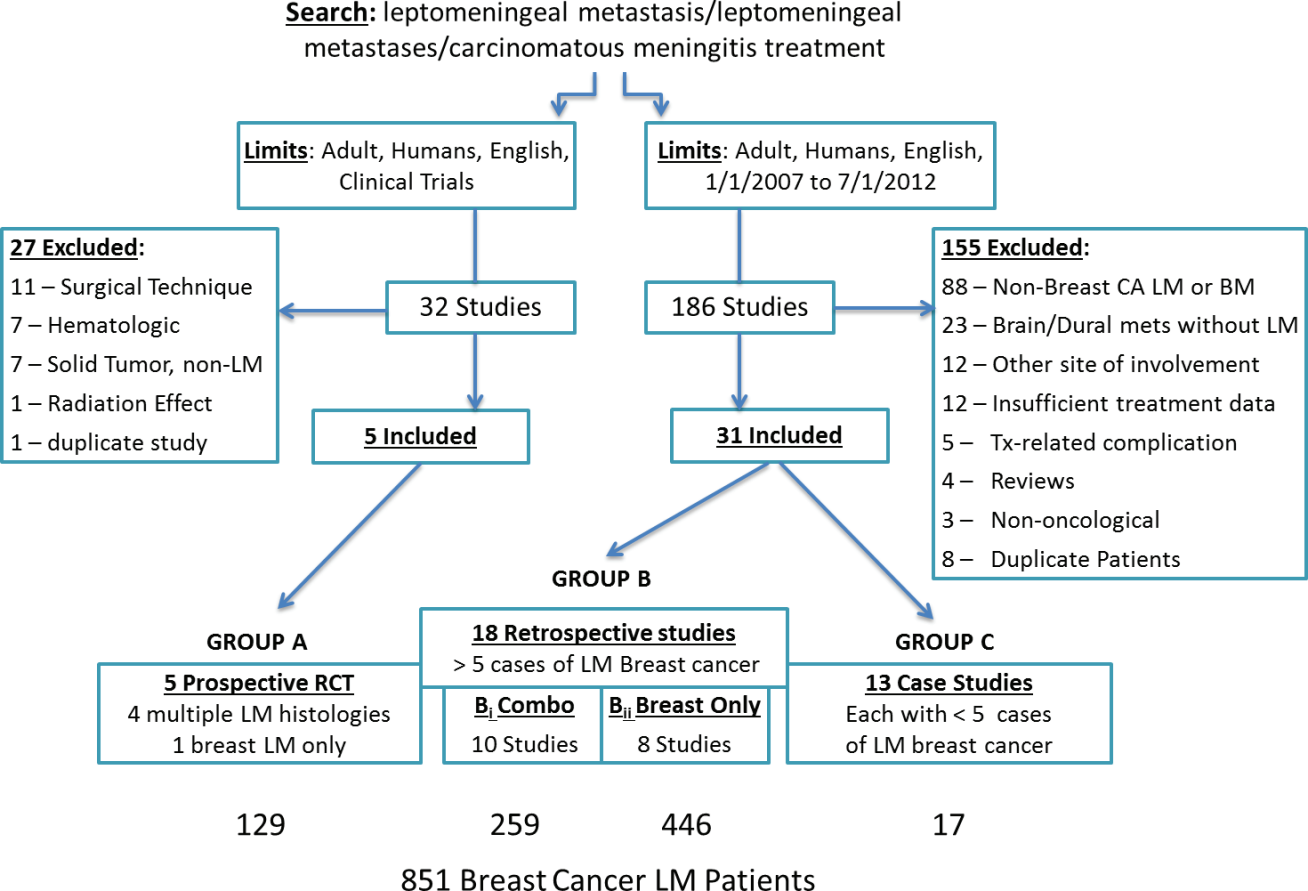
Search strategy with included and excluded studies used to identify the prospective trials, retrospective case series and case studies included for meta-analysis

The RCT search yielded 32 studies, of which 5 met the inclusion criteria. The second search yielded 186 studies, and 31 were included. One hundred eighty-two studies were excluded, and the reasons for exclusion are summarized in Figure 1. Studies were most commonly excluded because they did not include breast cancer LM. The 36 included studies resulted in a total of 851 breast cancer LM patients.

### GROUP A - prospective trials

Five trials were identified, with publication dates ranging from 1987-2010. Two were conducted in the US, [8, 9] one in Australia, [10] one in the Netherlands [11] and one was a multi-centered study in Europe and North America.[12] Route of chemotherapy as the primary outcome measure (intraventricular versus lumbar) was included in one study.[12] The remaining 4 compared intrathecal methotrexate (IT MTX) to an alternate treatment (IT liposomal cytarabine, [8] IT thiotepa, [9] IT MTX + cytarabine, [10] or intravenous MTX).[11] Four of the 5 studies reported multiple primary tumor types, and one [11] was breast cancer LM only.

There were 129 breast cancer LM cases out of a total of 300 LM cases (43%). None of the studies reported hormone receptor or HER-2 status. Breast cancer-specific survival data was available from 3 of these studies (71 patients). IT chemotherapy was given in 86%, and was associated with a median survival ranging from 9-18.3 weeks (*n* = 53; weighted mean 14.94 weeks). One study prospectively examined IV methotrexate with a median survival of 18.3 weeks (*n* = 18).[11]

### GROUP B - retrospective studies

#### Bi: multiple primary tumor types

There were 10 retrospective studies with a total of 693 LM patients, 259 (37%) from breast cancer. Four of these were descriptive, [13-15] 3 were single group treatment studies (topotecan plus ifosphamide, [16] continuous 5 day IT MTX [17] and whole-brain radiation therapy only [18]), and 3 made comparisons between treatment groups (reservoir on/off ventriculoperitoneal shunt versus Ommaya, [19] positive versus negative cerebrospinal fluid (CSF) cytology [20], and high versus low KPS.[21]

The weighted mean overall survival for the all of the breast cancer LM subjects in these studies was 15.3 weeks (*n* = 229; range 7-35 weeks). A Karnofsky Performance Status (KPS) less than 70 was associated with the shortest survival (7 weeks; *n* = 10), and KPS ≥70 was associated with a median survival of 29 weeks (*n* = 28). The 8 remaining studies did not use performance status as a criterion for exclusion. In these, the median overall survival ranged from 7.2-35 weeks (weighted mean 13.7; *n* = 191).

In the 8 studies with individual comparison data, survival in breast cancer LM (15 weeks; *n* = 145) was longer compared to other solid tumor LM (8.3 weeks; *n* = 131) and lung cancer LM (8.7 weeks; *n* = 83). The frequency of active systemic disease was slightly higher in solid tumor LM compared to breast cancer LM (84 versus 70%). Survival was longer for LM lymphoma compared to breast cancer (24.2 weeks; *n* = 55 versus 15.4 weeks; *n* = 146).

#### Bii: breast cancer only studies

Eight retrospective cohort studies of breast cancer LM with a total of 446 cases. Two of these reported all CNS metastasis in breast cancer (LM and parenchymal brain metastasis). There was also one prospective observational study, [22] and one retrospective subgroup analysis of a randomized treatment trial.[3] The Remaining 6 studies were institutional retrospective cohort studies of breast cancer LM exclusively.[23-28]

Ages ranged from 26-78 years, with a weighted mean age of 48.7 years. Two of the studies required positive CSF cytology for inclusion, [26, 27] while the others included LM cases diagnosed by either CSF or MRI findings. In studies that included initial CSF cytology, the composite sensitivity was 71.1% (range 67-75%; *n* = 322). The overall sensitivity of MRI at diagnosis was 77% (range 67-86; *n* = 349). Concurrent brain metastasis were present in 42.8% of cases (range 25-54.4%; *n* = 432), and 83.9% (58-100; *n* = 310) had active systemic cancer at the time of LM diagnosis.

The hormone receptor (HR) and human epidermal growth factor (HER)-2 status of the primary breast cancer was reported in 6 studies, however survival information related to molecular subtype was only available in 3.[23, 25, 28] HR positivity was observed in 48.1% (*n* = 156; range 35.3-58%), HER-2 positivity was seen in 27.2% (*n* = 235; range 15-40%), and triple negative breast cancer represented 27.6% (*n* = 290; range 21-36.8%). Negative HR status was associated with worse survival in one study.[25] HER-2 positive breast cancer treated with trastuzumab was found to have a longer time to LM compared to HER-2 positive non-trastuzumab treated individuals (15.2 versus 9.9 months; p = 0.008). [24] Triple negative breast cancer had a shorter interval between diagnosis and the development of LM compared to hormone receptor positive breast cancer (21.8 versus 64.6 months; p = 0.002) and an earlier age of onset (43 versus 50 years; p = 0.03).[24]

The most common treatment was intra-CSF chemotherapy, used in 86% (*n* = 410). Methotrexate was used as first line in 59% and liposomal cytarabine was used in 29% (12% unspecified). The number of treatments varied from 0-33, with a median ranging from 4-8.[23, 25, 27, 28] Clinical response to treatment was found in 68.5% (*n* = 330), and cytologic response was observed in 30.8% (*n* = 143).

Median time to LM diagnosis ranged from 17.9-88.8 months.[23, 25, 27, 28] The median LM overall survival for these studies was 18.1 weeks (range 11.3-35.2 weeks; *n* = 439). Composite 12 month survival was 13.6% (range 7.4-24.1%; *n* = 411). There were a number of individual factors that significantly impacted survival, and these are summarized in Table 1. Other factors that were examined but non-significant included age, [23] CSF protein and glucose levels, [23, 26] histology and HER-2 status.[25]

**Table 1:** Prognostic factors associated with survival in LM breast cancer

PROGNOSTIC FACTOR	FAVORABLE	UNFAVORABLE	NON-SIGNIFICANT
CLINICAL			
Initial Performance Status		[23]– ECOG >2 – HR 8.44; p<0.001 [25] - ECOG >2 – p=0.006 [27] – KPS <60 – HR 1.95; p 0.015	[26] – ECOG 0,1,2 versus 3,4 [24] – ECOG 0,1 versus 2,3,4
Histologic Grade		[23] - Grade 2 - HR 4.86; p=0.044 Grade 3 – HR 9.56; p=0.007	[25]
No Active Systemic Cancer	[24] p=0.035		[26]
Hormone Receptor Negative Primary		[25] – p=0.04	[23, 24, 28]
Triple Negative Primary		[28]	[24]
DIAGNOSTIC			
Elevated CSF Cyfra 21-1 level		[25] – p=0.009	
THERAPEUTIC			
Any chemo	[24]		
IT Chemo	[27] p=0.001		[23]
IV Chemo	[29] p<0.001		[23]
Combined Modality Tx	[24, 28] p=0.008		
> 3 prior chemotherapy regimens	[25]		
RESPONSE			
Clinical	[28]		
Progression after 1st cycle of treatment		[25] p<0.001	
Biological	[24] p=0.001 [25] p=0.003 [26] p=0.005		

ECOG= Eastern Cooperative Oncology Group; KPS= Karnofsky Performance Status; HR=Hazard Ratio; CSF= cerebrospinal fluid; cyfra = cytokeratin fragment; IT=intrathecal; IV=intravenous

### GROUP C - case studies

Among 13 case reports, there were 17 cases of LM breast cancer (range 1-3) (Table 2). The median age at diagnosis of those reported was 44 (*n* = 17; range 31-61). Spinal fluid cytology was positive in 76.9%. Hormone receptor status of the primary breast cancer was reported in 11 cases, with 54.5% hormone receptor positive. HER-2 positivity was reported in 73.3%. All of the HER-2 positive cases had received intravenous trastuzumab in the treatment of their systemic disease. Evidence of non-CNS metastasis was present in 88.2%, and brain metastasis were found in 64.7%. Whole brain radiation therapy was given to 70.6%.

**Table 2:** Summary of Group C Case studies - treatment regimens and median overall survival

Study	Publication Year	n	Treatment	Dosing	Age	median OS (weeks)
						
[30]	2011	2	IT Tras + IT MTX + IT Ara C	Trastuzumab 40mg weekly; MTX 15 mg; AraC 24 mg	43	58.7
			IT Tras + IT MTX + IT Ara C	Trastuzumab 100mg weekly; MTX 15 mg; AraC 24 mg	39	32
[31]	2009	1	Trastuzumab + capecitabine	trastuzumab IV + Capecitabine 1650mg/m2	44	43.4+
[32]	2008	1	IT Trastuzumab + IT MTX		48	7.9
[33]	2011	1	IT Trastuzumab	25mg weekly via LP x 67 treatments	44	117.3
[34]	2008	1	IT Trastuzumab	IT trastuzumab 20mg-100mg weekly	58	30.4
[35]	2008	1	RT + liposomal cytarabine	liposomal cytarabine 50mg every 14 days	55	52+
[36]	2009	2	liposomal cytarabine + TMZ	100mg/m2 TMZ + liposomal cytarabine every 14 days	43	41.3
					42	73
[37]	2009	1	liposomal cytarabine	liposomal cytarabine 50mg every 14 days	51	186.8+
[38]	2011	1	Lapatanib		45	12+
[39]	2007	1	Capecitabine+RT, IT MTX+ Ara C	IT MTX + Ara C 2 times per week x 5 doses	38	52
[40]	2007	3	Capecitabine		34	78
					54	52
					46	26
TOTALS		15			44	52

IT=intrathecal; IV=intravenous; MTX=methotrexate; Ara C= cytosine arabinoside; TMZ=temozolomide; RT=radiation therapy; mg=milligrams; m2=meters squared.

Treatment protocols varied greatly in this cohort (Table 2). IT chemotherapy (trastuzumab (6), methotrexate (5), liposomal cytarabine (5), or cytarabine (3)) was given to 70.6% either as a single agent (4) or as part of combination therapy (8). Standard dosing was used for IT methotrexate (15mg), cytarabine (24mg), and liposomal cytarabine (50mg). However, the dosing of IT trastuzumab ranged from 20mg to 100mg per treatment, and the frequency of treatments varied from weekly to dosing every 21 days. In some cases, capecitabine was given either alone (3), or in combination with other treatments (3). Lapatinib and intravenous trastuzumab were each reported once.

The median overall survival was 52 weeks (range 7.9-186.8+) for the group of case studies. Clinical responses were observed in 100% of cases.

## DISCUSSION

Albeit less common than other sites of metastasis, LM is a distinct form of metastatic spread, often occurring in individuals with advanced systemic breast cancer. Data to guide treatment decisions are limited, and it is difficult to draw conclusions based on the pooling of available data. Of the breast cancer cases identified in this review, in only 15% were data obtained prospectively. The prospective trials compared intrathecal methotrexate to different types or modes of delivery of chemotherapy. None of the prospective studies examined the impact of molecular subtype on survival outcome, and only 3 of the 5 reported breast cancer-specific survival. There were no studies that included a control arm, and thus the impact of intra-CSF chemotherapy versus radiation or no treatment cannot be determined based on these results. A mean overall survival of 15 weeks from the time of LM diagnosis in breast cancer when treated with intrathecal chemotherapy appears consistent across group A studies.

Mixed primary tumor retrospective series (Bi) studies facilitated comparison of survival between breast cancer and other solid tumor LM. Based on the pooled results, breast cancer LM survival (15 weeks) was nearly double that of lung cancer LM (8.7 weeks) and composite solid tumor LM (excluding breast CA) (8.3 weeks), but shorter than leptomeningeal lymphoma (24.2 weeks). Since these were not randomized, conclusions about the impact of treatment on survival could not be drawn.

The only information about hormone receptor status came from breast cancer only (Bii) retrospective series. These drew variable conclusions about the impact of molecular subtype on time to development of LM and the impact on survival (Table 1). No individual studies found an impact on survival based on HER-2 status. Conclusions from these are limited due to confounders inherent in retrospective methodology including a referral bias (the retrospective institutional series are completed at large regional cancer centers), and selection bias, as the cases reported are generally those with adequate functional status to undergo treatment with one or multiple lines of therapy.

There was a notable reporting bias in group C (case reports) reviewed (52 week survival with 100% clinical response), as well as considerable heterogeneity in treatment regimens. However, potentially active novel treatments such as intrathecal trastuzumab may be considered for more rigorous evaluation given the individual reports regarding safety and potential benefit.

High quality prospective trials with a more consistent approach to trial design and result reporting are necessary to determine the effectiveness of new treatment approaches and facilitate meaningful comparison between studies. For example, variability regarding whether a positive CSF cytology is an inclusion criterion affects the patient population, potentially impacts survival, and when not standardized limits or invalidates comparison across studies. Investigation of CSF dynamics (outflow obstruction studies and CSF opening pressure measurement) is not universally adopted, but is important, especially when assessing response and toxicity to intrathecal chemotherapy. Overall survival, and 6 and 12-month survival % are potentially useful outcome measures that are easily compared between studies, but these have not consistently been reported in all series. Clinical response and progression free survival are less reproducible. Accounting for variation in study populations among factors such as the extent of active systemic disease, brain metastasis and baseline functional status may impact survival.

A panel of experts from the Response Assessment in Neuro-Oncology workgroup reviewed all randomized controlled trials and concluded that there is a significant unmet need for guidelines for evaluating endpoints in LM both for clinical practice and research purposes.[29] Standard diagnostic and response criteria, and a focus on prospective disease site-specific (i.e. breast cancer only) investigations are the only way of determining the safety and effectiveness of targeted molecular or combination therapies to advance the treatment of LM breast cancer.

## MATERIALS AND METHODS

### Search strategy

A search using the terms: ‘leptomeningeal metastasis’ or ‘leptomeningeal metastases treatment’ or ‘meningeal carcinomatosis treatment’ was completed using PubMED/MEDLINE. The results were limited to humans, adults 19+, English language and a date range of 1/1/2007-7/1/2012 to capture contemporary studies given changes in treatments. The main search was supplemented with a search for ‘leptomeningeal metastasis’ and ‘carcinomatous meningitis’ in web of science, and a review of abstracts from the American Society of Clinical Oncology (ASCO) annual meetings from 2007-2012.

A second search was conducted using the above search terms, limited to randomized controlled trials and adults 19 + years old, over all available dates (1/1/1966-7/1/2012) to identify prospective studies.

### Inclusion/exclusion citeria

The search results were systematically reviewed and included or excluded based on the following criteria:

**Table:** 

Inclusion Criteria	Exclusion Criteria
Publication 1/1/07-7/1/12	No LM demographic or outcome data reported
Humans	Studies reporting BM with no LM-specific data
English language	Duplicate study populations
Breast and other solid tumor LM	

Abstracts from all studies that met initial inclusion criteria were reviewed individually, and studies that reported original data on LM outcomes were included. Longitudinal studies that followed individuals with breast cancer and reported the incidence of developing CNS metastasis were excluded unless they also reported detailed LM treatment and survival outcome data. Duplicate study populations were identified by reviewing the methods and identifying overlapping dates from the same institution or database. In these instances, the study with the largest number of patients was included.

### Categorization of included studies

Included studies were divided into groups as follows:

Group A: Prospective Trials - Any LM study in which data was collected and analyzed prospectively.
Group B: Retrospective Studies - Series of ≥ 5 consecutive LM cases retrospectively analyzed through an institution or database. These are reported as either Bi: combined LM histologies or Bii: breast cancer LM only.
Group C: Case Studies - Descriptive reports of <5 breast cancer LM cases not collected consecutively.

### Data collection

All included LM studies were reviewed in detail, and data specific to breast cancer LM was extracted using a standardized data collection form. Instances where individual breast cancer LM data was not reported were excluded from the breast cancer analysis.
